# Potential benefits of pharmacist intervention in the detection and therapy of atrial fibrillation

**DOI:** 10.1177/17151635211019963

**Published:** 2021-06-13

**Authors:** Lauren Adam

**Affiliations:** Lauren Adam is a student in the Faculty of Pharmacy and Pharmaceutical Sciences, University of Alberta, Edmonton, Alberta

## Impact of atrial fibrillation

The scope of practice of pharmacists has expanded greatly over the years, providing patients with increased access to care and improved health outcomes. Pharmacist intervention has been shown to result in enhanced care in studies evaluating pharmacist management of conditions such as heart failure^1^ and dyslipidemia.^2^ Atrial fibrillation (AF) poses unique challenges to the health care system, due to difficulties with diagnosis, potentially serious complications and a large economic burden. AF is the most common arrhythmia and has affected as many as 200,000 Canadians.^3^ Globally, AF has affected approximately 37,574 million, and in the next 30 years, its prevalence is predicted to increase by 66%.^4^ The Canadian Heart and Stroke Foundation has reported that AF imparts a 3 to 5-fold increased risk of ischemic stroke, with AF causing a quarter of all strokes in those 40 or older.^3^ This is a critical health care issue, as strokes resulting from AF are often more severe and debilitating than those due to other causes.^5^ Such patients require comprehensive medical management and sometimes do not ever regain the same level of functioning. The physical and cognitive disabilities resulting from AF-related strokes affect not only the patient but also the health care system as a whole. The mean cost of an AF hospitalization is estimated to be $4735, with a median admission duration of 5 days.^6^ Even more shocking is the reported cost of stroke management, rehabilitation and resulting decreased productivity in Canada, totalling a staggering $3.6 billion per year.^7^ Due to the devastating health and quality-of-life outcomes, in addition to the economic implications, employment of further stroke prevention strategies is of the utmost importance.

## How can pharmacists help?

Implementation of arrhythmia screening by pharmacists in the community setting could greatly reduce the physical and financial burden of AF, resulting in improved quality of life and reduced mortality for patients. Pharmacists can play a vital role in the identification and management of this patient population, through facilitation of early detection practices and regular monitoring of anticoagulation therapy.

Early detection through regular screening could expedite the timely implementation of proper management, potentially preventing detrimental outcomes. In 2014, an innovative mobile single-lead electrocardiogram (ECG) device called AliveCor was developed and, with it, the opportunity to screen for AF in remote settings, such as community pharmacies. As the most accessible health care professionals, pharmacists could play a key role in assisting patients presenting with paroxysmal AF to obtain a proper diagnosis by recording their ECG within the pharmacy itself. The device is easy to use, is portable and produces a quality ECG heart rhythm recording in as little as 30 seconds.^8^ Patients must simply place their fingers on the sensor bar, and the device will produce a reading, with interpretation. This result, which is readily available in PDF format, can then be sent to the other health care professionals managing their care.

Multiple studies to date have examined the accuracy and reliability of the AliveCor system (AliveCor, Inc., United States). Recently, a systematic review by Hall et al.^9^ was published, which compared the results of 11 studies investigating the use of this novel ECG device. They assessed the feasibility and accuracy of the AliveCor system and presented their recommendations regarding its utility in community AF screening. While they found the sensitivity and specificity of the device to be variable, this heterogeneity was determined to be attributable to differences in study populations examined, prevalence of AF risk factors (such as the presence of chronic diseases or older age) within these groups, and the methods followed (single-point-in-time recording vs intermittent recordings) in the included studies. Of the research they examined, some studies found the sensitivity of this device to be >98% and the specificity to be >99%.^9^

National Winner 2020

Capsi Student Literary Challenge

## Addressing current care gaps

Pharmacists can also play a vital role in ensuring patients diagnosed with AF are initiated and maintained on proper oral anticoagulant (OAC) therapy in the community. Research has demonstrated a 70% to 80% reduction in risk of stroke with the use of OAC therapy in this patient population.^3^ However, despite the demonstrated benefits, research has shown OAC medications to be underprescribed in patients with AF. The PINNACLE Registry, cardiology’s largest outpatient quality improvement registry, found that as many as 50% of high-risk AF patients (CHADS_2_ score >3 or CHA_2_DS_2_-VASc score >4) were not prescribed OAC therapy.^10^ In patients initiated on OAC therapy, research has also shown that subtherapeutic dosing is common, with 1 study finding up to 20% of participants to be on inappropriate OAC doses for AF.^11^ Appropriate dosing is determined through consideration of the patient’s weight, renal function, age, bleeding risk and concomitant drug therapies,^11^ which provides an explanation for the increased frequency of inappropriate doses prescribed. This only further exemplifies why involving pharmacists more comprehensively in the management of patients with AF may help alleviate current gaps in care.

## Recommendations

Hall et al.^9^ determined the most cost-effective means of implementing pharmacy AF screening using AliveCor ECG technology to be through prioritization of patient populations at greatest risk. It has been demonstrated that factors such as hypertension, diabetes, tobacco and alcohol use, obesity and obstructive sleep apnea can increase the chances of developing AF.^3,4^ Advanced age appears to be one of the greatest nonmodifiable risk factors, with an estimated lifetime risk of AF development between 22% and 26% for those aged 40 to 55 years.^12^ Therefore, screening programs targeting older individuals with at least one potential risk factor may be the most feasible strategy for busy pharmacies to realistically provide this service, while maximizing the likelihood of identifying the presence of arrhythmia in undiagnosed patients.

## Conclusion

Research has shown that screening for AF using the AliveCor system can be performed easily, safely and with relatively good reliability in the community setting. By involving pharmacists more actively in the identification and monitoring of patients with AF, it may be possible to mitigate some of the deleterious impacts of this increasingly common condition through early detection and implementation of appropriate OAC therapy. ■
